# Supplementary material for: Transgenic interleukin 11 expression causes cross-tissue fibro-inflammation and an inflammatory bowel phenotype in mice
Source: PLoS One. 2020 Jan 9;15(1):e0227505. doi: 10.1371/journal.pone.0227505 (PMC6952089; doi:10.1371/journal.pone.0227505)
Supplement: S1 Table — (DOCX) [file pone.0227505.s001.docx]

| **S1 Table. Genotyping primers** | |  |  |
| --- | --- | --- | --- |
| **Primer** | **Sequence (5’-3’)** | **Annealing Temperature (℃)** | **Product size (bp)** |
| **Set 1: *Myh11-Cre*** | | | |
| Myh11cre F | TGA CCC CAT CTC TTC ACT CC | 65 | 287 |
| Myh11cre R | AGT CCC TCA CAT CCT CAG GTT |  |  |
| Internal positive control F | CAG CCA ACT TTA CGC CTA GC | 65 | 180 |
| Internal positive control R | TCT CAA GAT GGA CCT AAT ACG G |  |  |
| **Set 2: *Rosa26-Il11*** | | | |
| COO1 F | GTT TTG GAG GCA GGA AGC ACT TGC | 65 | 727 |
| KIn-ROSAGX6044 F | GCA GTG AGA AGA GTA CCA CCA TGA GTC C | 65 | 270 |
| COO1 R | CAATGCTCTGTCTAGGGGTTGGATAAGC | 65 |  |
