# Supplementary material for: Transgenic interleukin 11 expression causes cross-tissue fibro-inflammation and an inflammatory bowel phenotype in mice
Source: PLoS One. 2020 Jan 9;15(1):e0227505. doi: 10.1371/journal.pone.0227505 (PMC6952089; doi:10.1371/journal.pone.0227505)
Supplement: S2 Table — (DOCX) [file pone.0227505.s002.docx]

| **S2 Table. RT-qPCR primers** | | |
| --- | --- | --- |
| **Gene** | **Forward primer (5’ to 3’)** | **Reverse primer (5’ to 3’)** |
| *Col1a1* | GGGGCAAGACAGTCATCGAA | GTCCGAATTCCTGGTCTGGG |
| *Col1a2* | CCCAGAGTGGAACAGCGATT | ATGAGTTCTTCGCTGGGGTG |
| *Col3a1* | ATGCCCACAGCCTTCTACAC | ACCAGTTGGACATGATTCACAG |
| *Fn1* | CACCCGTGAAGAATGAAGA | GGCAGGAGATTTGTTAGGA |
| *Timp-1* | GGGCTAAATTCATGGGTTCC | CTGGGACTTGTGGGCATATC |
| *Mmp2* | ACAAGTGGTCCGCGTAAAGT | AAACAAGGCTTCATGGGGGC |
| *Il6* | AGGATACCACTCCCAACAGACC | AGTGCATCATCGTTGTTCATACA |
| *Ccl2* | GAAGGAATGGGTCCAGACAT | ACGGGTCAACTTCACATTCA |
| *Ccl5* | GCTGCTTTGCCTACCTCTCC | TCGAGTGACAAACACGACTGC |
| *Gapdh* | CTGGAAAGCTGTGGCGTGAT | GACGGACACATTGGGGGTAG |
