# Supplementary figures and images for: Transgenic interleukin 11 expression causes cross-tissue fibro-inflammation and an inflammatory bowel phenotype in mice
Source: PLoS One. 2020 Jan 9;15(1):e0227505. doi: 10.1371/journal.pone.0227505 (PMC6952089; doi:10.1371/journal.pone.0227505)

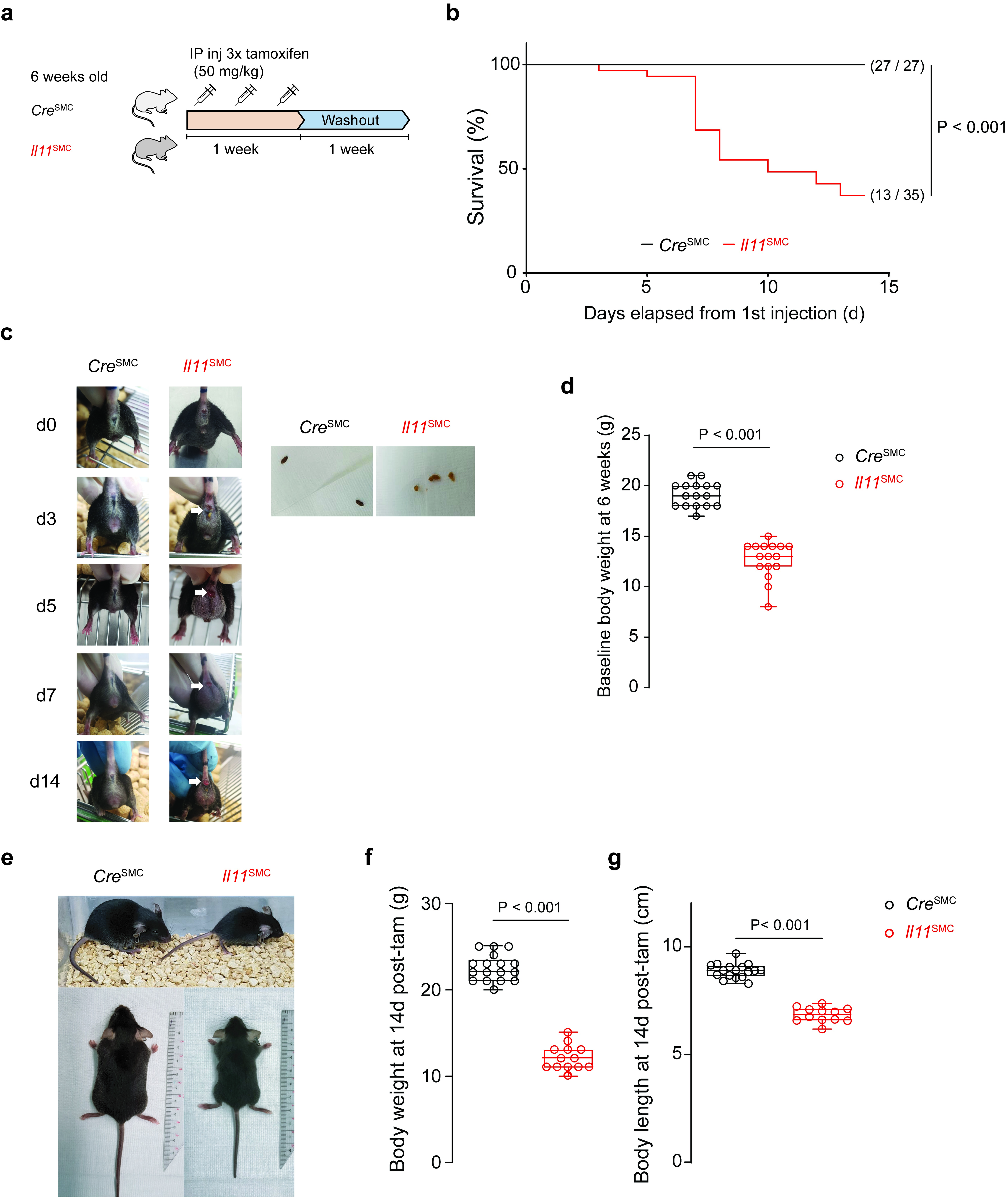

Supplement: S1 Fig — (a) Schematic diagram demonstrating the tamoxifen (tam) injection procedure in 6-week-old Il11SMC and CreSMC mice. (b) Survival curve of tam-treated Il11SMC (n = 35) compared to CreSMC mice (n = 27) mice from 1st injection starting at 6 weeks of age. Survival curves were compared with the log-rank Mantel-Cox test. (c) Representative images of the CreSMC and Il11SMC mice before (d0) and up to 14 days (d14) post-tam initiation (left). Note the presence of pale and loose stools in Il11SMC mice (right). The presence of rectal prolapse is indicated with white arrows. Tam-treated Il11SMC images presented here are different from Fig 1c. Images were not taken to scale. (d) Baseline body weight of 6-week-old CreSMC and Il11SMC mice before induction (n = 16 per group). Statistical analyses by two-tailed unpaired t-test; data expressed as median ± IQR, whiskers represent the minimum and maximum values. (e) Representative images of CreSMC and Il11SMC mice at d14 post-Tam initiation. (f) Collated body weights and (g) body lengths of tam-treated CreSMC and Il11SMC mice measured at d14 post-Tam initiation. (n = 12–17 per group). Statistical analyses by two-tailed unpaired t-test; data expressed as median ± IQR, whiskers represent the minimum and maximum values. (TIF) [file pone.0227505.s003.tif]

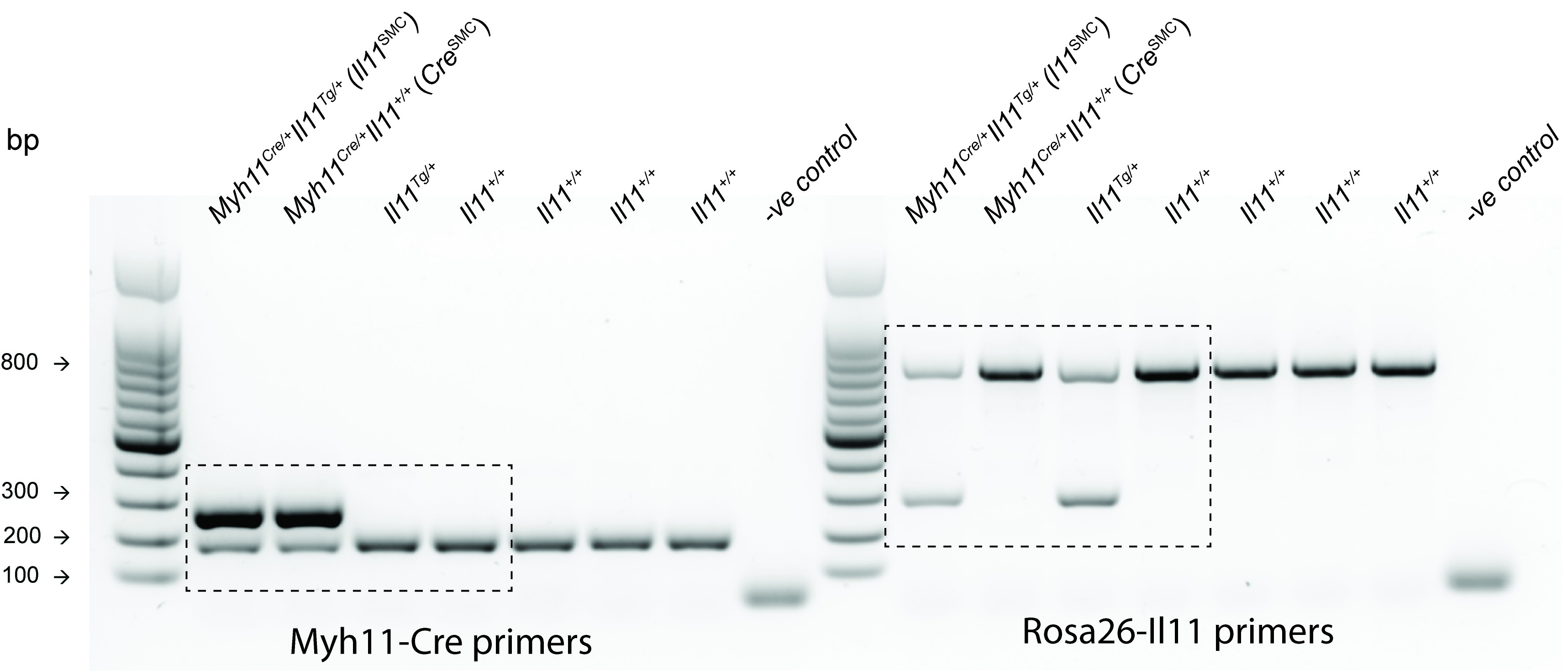

Supplement: S2 Fig — PCR products of DNA extracted from tail biopsies of 21-day-old mice by use of set primers for Myh11-Cre (left) and Rosa26-Il11 (right) (primers as listed in S1 Table) and analyzed by agarose gel electrophoresis. Dashed boxes indicate cropped blots used in Fig 1c. (TIF) [file pone.0227505.s004.tif]

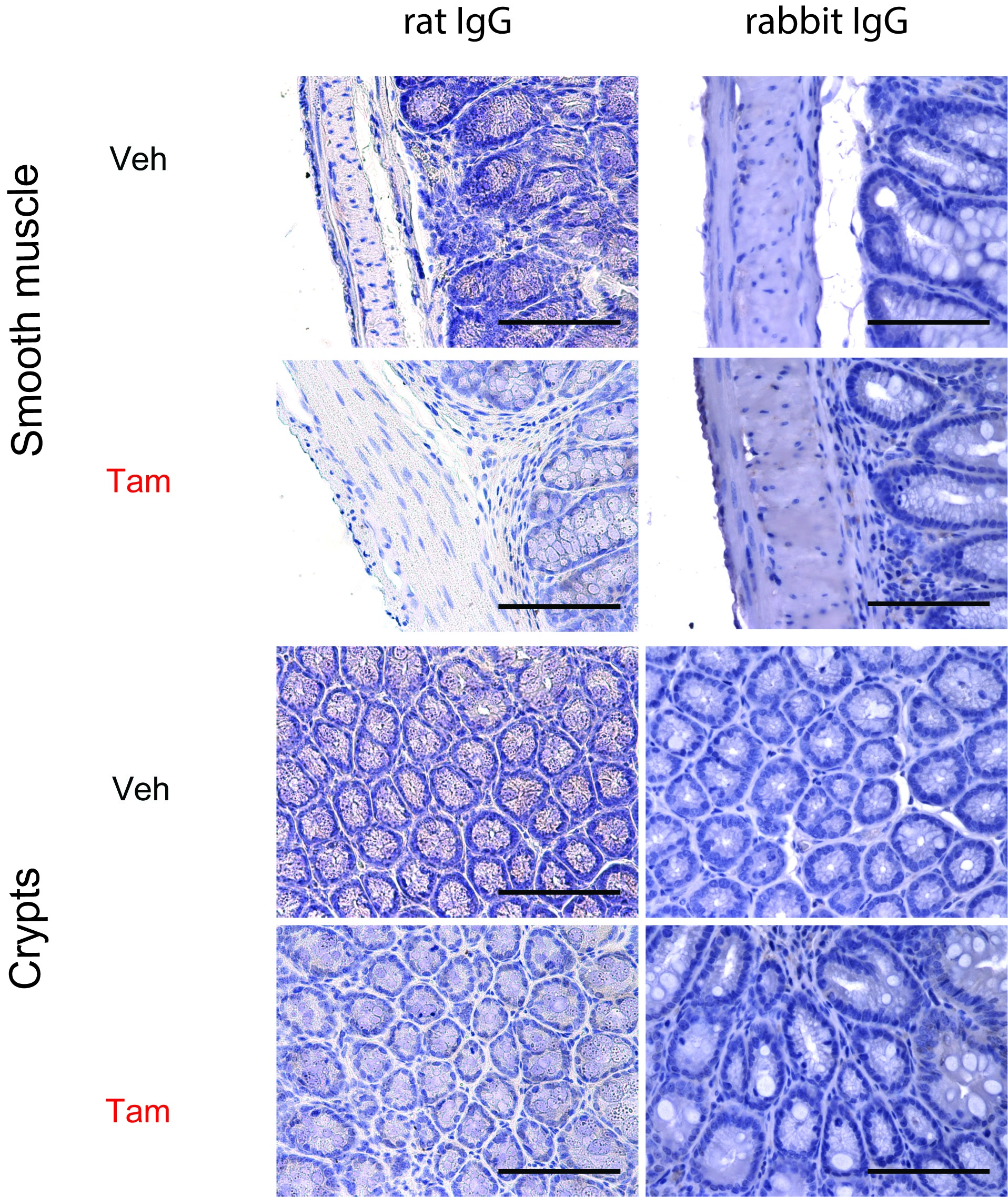

Supplement: S3 Fig — Smooth muscle and crypt staining with rat and rabbit IgG isotype controls demonstrate no positive staining in both veh- and tam-treated Il11-Tg colon. Scale bar represents 100 μm. (TIF) [file pone.0227505.s005.tif]
